# Epidermal Micromorphology and Mesophyll Structure of *Populus euphratica* Heteromorphic Leaves at Different Development Stages

**DOI:** 10.1371/journal.pone.0137701

**Published:** 2015-09-10

**Authors:** Yubing Liu, Xinrong Li, Guoxiong Chen, Mengmeng Li, Meiling Liu, Dan Liu

**Affiliations:** 1 Key Laboratory of Stress Physiology and Ecology in Cold and Arid Regions of Gansu Province, Cold and Arid Regions Environmental and Engineering Research Institute, Chinese Academy of Sciences, Lanzhou, 730000, P. R. China; 2 Shapotou Desert Research & Experiment Station, Cold and Arid Regions Environmental and Engineering Research Institute, Chinese Academy of Sciences, Lanzhou, 730000, P. R. China; Huazhong university of Science and Technology, CHINA

## Abstract

Leaf epidermal micromorphology and mesophyll structure during the development of *Populus euphratica* heteromorphic leaves, including linear, lanceolate, ovate, dentate ovate, dentate rhombic, dentate broad-ovate and dentate fan-shaped leaves, were studied by using electron and light microscopy. During development of heteromorphic leaves, epidermal appendages (wax crystals and trichomes) and special cells (mucilage cells and crystal idioblasts) increased in all leaf types while chloroplast ultrastructure and stomatal characters show maximum photosynthetic activity in dentate ovate and rhombic leaves. Also, functional analysis by subordinate function values shows that the maximum adaptability to adverse stress was exhibited in the broad type of mature leaves. The 12 heteromorphic leaf types are classified into three major groups by hierarchical cluster analysis: young, developing and mature leaves. Mature leaves can effectively obtain the highest stress resistance by combining the protection of xerophytic anatomy from drought stress, regulation of water uptake in micro-environment by mucilage and crystal idioblasts, and assistant defense of transpiration reduction through leaf epidermal appendages, which improves photosynthetic activity under arid desert conditions. Our data confirms that the main leaf function is differentiated during the developing process of heteromorphic leaves.

## Introduction

During the entire life cycle of higher plants, they are repeatedly subject to diverse environmental stress, especially in desert ecosystems. Plant response to such unfavorable growth conditions can be a complex combination of physiological activity, individual morphology and long-term adaptive strategy [1, 2]. Leaves are exposed to aerial conditions more than any other plant organs, and changes in leaf characters have been interpreted as adaptations to specific environments [3]. Desert plants with strong drought-resistance often form special leaf dissection structures of pattern with special functions adapted to adverse circumstances [4].

*Populus euphratica* is a pioneer tree and a natural protection shield for the desert forest ecosystem in northwestern China [5, 6], praised as "hero tree of desert" due to characters tolerant to drought, sandstorms and salt. Thus, *P*. *euphratica* is considered an ideal plant species to study the mechanisms responsible for survival of woody plants under adverse environmental conditions in deserts [7]. Previous studies have provided useful information for understanding the adaptation mechanism of *P*. *euphratica* to abiotic stress, including eco-hydrological process of the forest community [8], photosynthetic and physiological characteristics of the tree [9–12], morphological and structural characters of the leaf [13], gene and protein response to stress on a molecular level [7, 14, 15]. Recently, scientists have succeeded in unraveling the whole genome sequence of *P*. *euphratica* and the genetic bases underlying the mechanism against salt stress [16].

On the other hand, we have noticed that *P*. *euphratica* is one of a few tree species which have developed heteromorphic leaves. There is a distinct leaf shape polymorphism from the lower to upper crown of the tree. The leaf shapes of adult *P*. *euphratica* vary from linear to dentate broad-ovate, and the three typical heteromorphic leaves are lanceolate, ovate and broad ovate [17]. The study of Li and Zheng [18] shows that the structural characteristics of the diversiform-leaves of *P*. *euphratica* are related to its eco-adaptability. Electrophoresis analysis of the protein expression indicates that the regulated gene expression in heteromorphic leaves of *P*. *euphratica* results in the generation of different leaf shapes, in order to adapt to the local environment [19]. Studies on stomatal characteristics and photosynthesis of polymorphic *P*. *euphratica* leaves indicate that leaf shape, anatomic structures and photosynthetic characters change during leaf development [17]. PSII activity is related to the water content in three typical heteromorphic leaf types of *P*. *euphratica* trees [20] and the broad-ovate leaves exhibit C_4_-like enzymological features [21]. However, these studies focused on the three typical heteromorphic leaf types. There are more than 10 diversiform-leaf types in a mature *P*. *euphratica* tree, and the shape change is gradual and continuous throughout the developmental process. A systematic study is lacking on the development of epidermal micro-morphological and mesophyll structures of heteromorphic leaves at different development stages, especially for adaptation characters from quantitative to qualitative change of *P*. *euphratica* heteromorphic leaves in natural communities.

In the present study, an experiment was designed to answer the following question: for a given natural habitat in the National Natural Reserve of *P*. *euphratica* in the Ejina Oasis in the lower reaches of the Heihe River, northwestern China, how to evaluate the functional differentiation and stress resistant ability at quantitative level of leaf micromorphology and structure at different development stages of heteromorphic leaves? To efficiently evaluate the focused function and the integration ability to stress tolerance of *P*. *euphratica* heteromorphic leaves, it is necessary to establish quantitative criteria that can be numerically addressed rather than qualitatively assessed in leaf characters. Clustering features and subordinate function values of the heteromorphic leaves were used as selection indicators evaluating the capability of stress tolerance. Twelve heteromorphic leaves were selected according to the dentate degree during leaf development, from bottom to top in the crown of one *P*. *euphratica* tree. Leaf epidermal micromorphology and mesophyll structures at different development stages of heteromorphic leaves were analyzed to elaborate the leaf trait values, in order to further explore the long-term adaptive strategy in desert conditions.

## Material and Methods

### Ethics Statement

*P*. *euphratica* is listed as a protected species in the desert forest ecosystem of northwestern China. Our study was performed at the National Natural Reserve of *P*. *euphratica* in the Ejina Oasis, lower reaches of the Heihe River, northwestern China. This work belongs to the Major Research plan of the National Natural Science Foundation of China entitled ‘Integration research on eco-hydrological process of Heihe River basin’, and was permitted by the local forest administration named ‘*Populuse uphratica* Forest Administration in Ejina Oasis National Natural Reserve’.

### Plant species and sampling site

This experiment was conducted at the National Natural Reserve of *P*. *euphratica* (41°59.064' N, 101°07.456' E; elevation: 928 m) in the Ejina Oasis in the lower reaches of the Heihe River, northwestern China (S1 Fig). *P*. *euphratica* is the dominant native woody species, capable of forming an imposing canopy (>20 m tall) in some areas (S2 Fig). Weather and vegetation conditions are reported in Cao *et al*. [22]. This area has an arid continental climate and is one of the extreme arid regions in China. Annual precipitation in the region is < 50 mm, of which 84% occurs during the rainy season (May–September); evaporation is > 3,500 mm. Average yearly air temperature is approximately 8.2°C. Prevailing wind directions are northwest in winter and spring, and southwest to south in summer and autumn. Yearly average wind speed is approximately 3.4–4.0 m s^-1^.

### Materials selected

**Fig 1:**
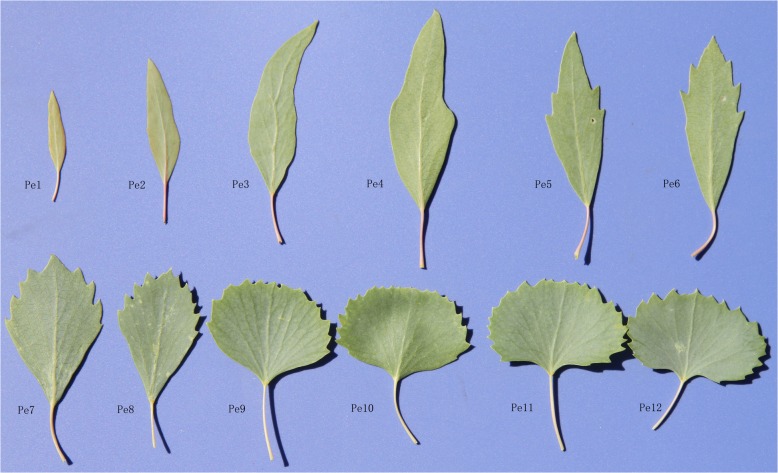
Heteromorphic leaves of one *Populus euphratica* tree from bottom to top of the crown (Pe1–Pe12) at the *P*. *euphratica* Reserve in the Ejina Oasis. Pe1, linear leaf; Pe2, Pe3, lanceolate leaf; Pe4, ovate leaf; Pe5, Pe6, dentate ovate leaf; Pe7, Pe8, dentate rhombic leaf; Pe9, Pe10, dentate broad-ovate leaf; Pe11, Pe12, dentate fan-shaped leaf. From Pe5 to Pe12, the level of dentate degree increased.

During the growth and development of a *P*. *euphratica* tree, leaf morphology changes accordingly, with sequential development of linear, lanceolate, ovate, or broadly ovate leaves. In one tree with numerous and simultaneous types of leaf shapes, the heteromorphic leaf distribution in the canopy from top to bottom is broadly ovate, ovate, lanceolate or linear, respectively. We selected all types of heteromorphic leaves according to the dentate degree located from bottom to top in the crown of one healthy *P*. *euphratica* tree, named as Pe1 to Pe12, illustrated in Fig 1. All leaf samples were collected on May 26, 2012. The leaves were cut into 0.5×0.5 cm^2^ sections after 0.5 cm segment from the leaf tip. 10–12 slices of each heteromorphic leaf type were selected, and then immediately fixed in a phosphate buffer (pH 7.2) containing 3% glutaraldehyde for 24 h.

### Leaf epidermal micromorphology and anatomy analyzed by electron and light microscopy

Sample preparation method is according to Liu *et al*. [23]. Samples were washed twice for 10 min in phosphate buffer (0.1 M) at pH 7.2, and then post-fixed with 1.0% OsO_4_ in the same buffer overnight. After post-fixing, samples were washed with the same phosphate buffer and dehydrated in increasing concentrations of ethanol (30, 50, 70, 96, 100%). Pre-inclusion of samples in freshly prepared resin at 35°C (24 h) were followed by final inclusion in acetone/epon812 (1:1) (12 h at 45°C and 24 h at 65°C).

For light microscopy, semi-thin sections (1.0 μm) were stained with 1% toluidine blue and observed. Photographs were taken with a Zeiss Q500 IW light microscope, and digital images were captured using a BX41-32P02+MD20 camera. Three independent experiments were performed for each staining analysis. To determine the cross-sectional area of different tissues, 4–6 different leaf slices were analyzed with an image analyzer (ImageJ Launcher-1.4.3.67).

For scanning electron microscopy (SEM), samples were fixed, post-fixed and dehydrated as above, dried using JFD-310 apparatus (Japan), covered with 20 nm of gold, and observed in a JSM-6380 (Japan) microscope. Images were analyzed with an image analyzer (Smile View-6.31.100.1190).

For transmission electron microscopy (TEM), 3–5 ultra-thin sections (70 nm) from different leaves were obtained using an ultra-microtome (LKB-V, Sweden) and stained with 3% uranylacetate followed by lead citrate before observation with a TEM (JEM-1230, Japan), and images were analyzed with an image analyzer (Smile View-6.31.100.1190).

### Morphological and anatomical traits measurement

In this study, a total of ten traits related to stress resistance were selected for investigation. Definition of traits and their description of measurements are listed in Table 1. These traits included four epidermal micromorphological traits and six mesophyll anatomical traits, which represented various criteria used for stress tolerance selection of *P*. *euphratica* leaves. The selected ten characteristics are recognized as primary traits related to stress resistance according to references noted on each trait in Table 1.

**Table 1:** The main characteristics related to stress resistance of the *Populus euphratica* leaves.

Classification of traits	Trait names and description of their measurement
Epidermal micromorphological traits	**Stomata density (SD, number/mm** ^**2**^ **)** [46], the average number of stomata per square millimeter on the adaxial surface of ten random leaf splices. The number of stomata in a visual field was measured by scanning electron microscopy and converted to number per square millimeter.
	**Stomata size (SS, μm)** [46, 47, 48], the mean value of stomata length and stomata width of 15 individual stomata. Stomata length and width were measured by scanning electron micrographs using an image analyzer, and the average size of the stomata was calculated on the adaxial surface of ten random visual field on five leaf slices.
	**Trichomes coverage (TC, %)** [40, 42, 43, 44], the average value of area percentage of trichomes covering the adaxial leaf surface of ten random leaf slices. Area percentage of trichomes was the integral area ratio scanned on the scanning electron micrographs.
	**Cuticular wax coverage (CWC, %)** [25, 36], the average value of area percentage of cuticular wax covering the adaxial leaf surface of ten random leaf slices. The area percentage was the integral area ratio of cuticular wax scanned on the scanning electron micrographs.
Mesophyll anatomical traits	**Lamina thickness (LT, mm)** [25], the average thickness of the middle part of leaf measured by light microscopy at 40× magnification in the leaf cross-section of ten random leaf slices.
	**Ratio of epidermal cell thickness to lamina thickness (REL)** [25], epidermal cell thickness was the average thickness measured by light microscopy at 40× magnification in the leaf cross-section. Ratio of epidermal cell thickness to lamina thickness was the average value of ten random individual leaf slices.
	**Ratio of palisade tissue to lamina thickness (TCR)** [25], the average data of palisade tissue thickness to lamina thickness of ten random individual leaf slices. Palisade tissue thickness was also measured by light microscopy at 40× magnification in the leaf cross-section.
	**Cuticular wax thickness (CWT, μm)** [36], the average thickness of cuticle wax of ten random visual fields of five leaf slices. Cuticular wax thickness was measured by transmission electron microscopy in the leaf cross-section.
	**Number of crystal idioblasts (NC, number/ 100 cells)** [26, 28, 29], the average number of crystal idioblasts per 100 mesophyll cells of ten random leaf slices. The number of mucilage cells and mesophyll cells in a visual field was measured by light microscopy at 40×magnification in the leaf cross-section.
	**Number of mucilage cells (NM, num/ 100 cells)** [32, 35], the average number of mucilage cells per 100 mesophyll cells of ten random leaf slices. The number of mucilage cells and mesophyll cells in a visual field was measured by scanning electron microscopy in the leaf cross-section.

Abbreviations of the main traits are same as in the following tables. These selected primary traits related to stress resistance are recognized according to references noted on each trait.

### Data processing and statistical analysis

Data were arranged using Microsoft Excel 2010 for statistical analysis. The significance of the differences in average values of ten individual measurements for ten traits of morphological and anatomical study between *P*. *euphratica* heteromorphic leaves were evaluated by means of the one-way analysis of variance (ANOVA, P < 0.05) using Data Processing System software (SPSS, Chicago, IL, USA). To classify and discriminate among the leaf samples, hierarchical cluster analysis (HCA) was performed according to the method of Yi *et al*. [24] based on the maximum similarities with SPSS software.

### Leaf functional traits of stress resistance

Leaf functional traits were assessed by subordinate function value. Subjection function value of the major traits of each type of heteromorphic leaves was determined by the following Eq (1):

$$\text{R}\left({\text{X}}_{\text{i}}\right)=\left({\text{X}}_{\text{i}}–{\text{X}}_{\text{imin}}\right)/\left({\text{X}}_{\text{imax}}–{\text{X}}_{\text{imin}}\right)\;\left(i=1,2,3,\ldots ,n\right)$$ (1)

where, R(X_i_) is subordinate function value of *i* trait of each type, X_i_ is trait score of each type, X_imax_ and X_imin_ is the maximum and minimum of *i* trait among all types, respectively. When the value of traits was negative correlated to stress resistance, subjection function value was determined by the replacement Eq (2):

$$\text{R}\left({\text{X}}_{\text{i}}\right)=1-\left({\text{X}}_{\text{i}}–{\text{X}}_{\text{imin}}\right)/\left({\text{X}}_{\text{imax}}–{\text{X}}_{\text{imin}}\right)\;\left(i=1,2,3,\ldots ,n\right)$$ (2)

Stress resistance of each type of heteromorphic leaves was graded using the major traits according to their means of subordinate function values.

## Results

### Cross-section anatomical characteristics

**Fig 2:**
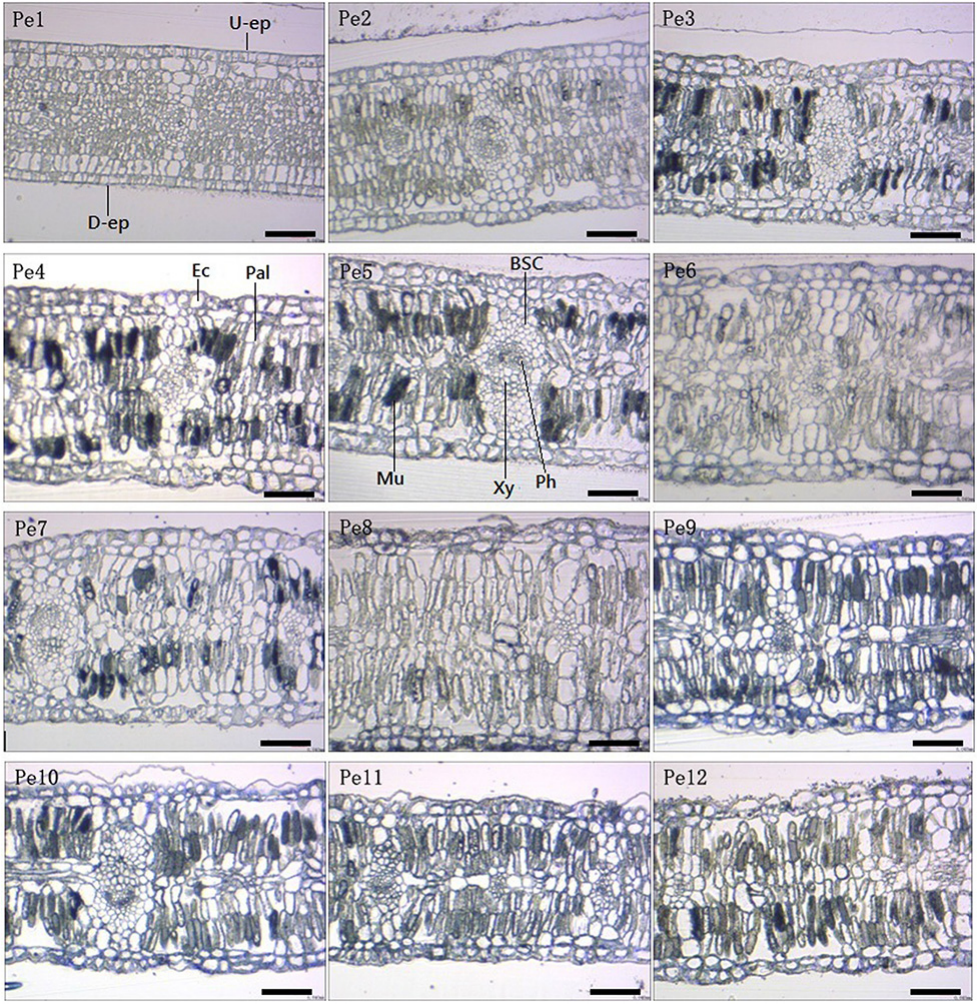
Cross-section anatomical characteristics of *Populus euphratica* heteromorphic leaves from Pe1 to Pe12. The images are shown in ×40 magnification, scale bars = 10 μm. U-ep, up-epidermis; D-ep, down- epidermis; Ec, epidermal cells; Pal, palisade tissue; BSC, bundle sheath cells; VB, vascular bundle; Mu, mucilage cells.

**Fig 3:**
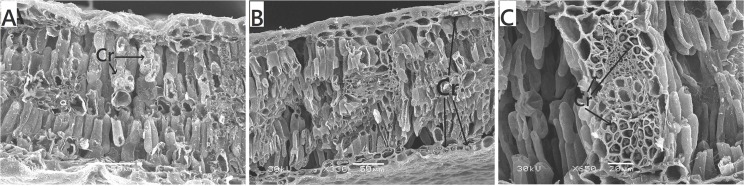
Crystal idioblasts exhibited in the cross-section of *Populus euphratica* heteromorphic leaves. The images show that crystals appear in all parts of the leaf structure, including (A) palisade tissue, (B) epidermal cells, and (C) Phloem of vascular bundles. Scale bars: A, B = 50 μm, C = 20 μm. Cr, crystal cells.

*P*. *euphratica* leaves are isobilateral from the cross-section anatomy (Fig 2). Two layers of epidermal cells are exhibited in both adaxial and abaxial surfaces. The cylindrical or ellipsoid cells are closely arranged in the palisade tissue, and no spongy tissue was observed in the mesophyll besides the vascular bundle systems. Pe1 materials were young leaves with square epidermal cells regularly arranged in the epidermis. In leaves from Pe3 to Pe12, the irregular epidermal cells with thick cell walls were filled with mucilage or crystals (Figs 2 and 3). Also, numerous mucilage cells appeared in the palisade tissue and the quantities of mucilage cells increased from Pe3 to Pe12 (Table 2). The leaf cross-section scanned by SEM show that crystal idioblasts appeared in all parts of the leaf structure, including palisade tissue, epidermal cells, and phloem of vascular bundles (Fig 3). The number of crystal idioblasts (NC) increased significantly from Pe1 to Pe12 (Table 2).

**Table 2:** Statistical data on epidermal micromorphological and mesophyll anatomical characters of the *Populus euphratica* heteromorphic leaves.

Mn	SD	SS	TC	CWC	LT	REL	TCR	CWT	NC	NM
Pe1	159.8571±9.71a	12.47857±1.05a	0a	87.42857±6.54a	0.373±0.08a	0.16622±0.02a	0.43208±0.05a	0.110917±0.01a	45.16±2.31a	0a
Pe2	179.32±10.33b	14.4±1.14b	0a	87.71429±7.19a	0.4444±0.07b	0.175068±0.03b	0.530828±0.07b	0.682±0.07b	53.26±2.64a	2.68±0.15b
Pe3	178.5714±10.21b	15.02857±1.27b	0a	92.57143±6.91b	0.4448±0.12b	0.210432±0.03c	0.524955±0.05b	0.97175±0.08b	55.34±2.18a	9.51±0.53c
Pe4	183±10.56b	15.70714±0.97b	0a	94.71429±8.22b	0.5038±0.11c	0.19214±0.04b	0.449682±0.06a	0.742167±0.08b	58.48±3.22b	12.45±0.72c
Pe5	175.8517±9.64b	16.43571±1.34b	0a	95.57413±7.94b	0.4904±0.09c	0.165987±0.03a	0.532898±0.05b	0.734083±0.07b	60.67±3.78b	12.66±0.86c
Pe6	172.1429±9.57b	17.32857±1.67bc	0a	94.2871±8.56b	0.5252±0.13d	0.180503±0.02b	0.548681±0.06b	1.153583±0.09c	62.42±3.82b	14.22±0.88c
Pe7	172.2857±10.12b	17.29286±1.28bc	0a	92.1455±7.82b	0.5126±0.09d	0.188022±0.03b	0.547879±0.07b	1.073±0.08b	62.86±3.76b	15.18±0.96c
Pe8	180.4286±11.34b	19.05714±1.62c	3.57±0.24b	93.85714±6.85b	0.5914±0.16e	0.150152±0.01a	0.521926±0.05b	1.12354±0.09c	68.13±4.12c	17.26±0.83d
Pe9	182.2857±10.68b	18.49286±0.96c	9.14±0.37d	94.81325±7.24b	0.508±0.11c	0.185827±0.04b	0.594258±0.07c	1.282333±0.11c	70.25±4.34c	18.64±0.97d
Pe10	178.7148±9.48b	19.15174±1.55c	13.54±0.39e	95.71426±8.05b	0.5022±0.15c	0.156113±0.02a	0.595713±0.08c	1.185417±0.09c	71.08±4.61c	20.22±1.02d
Pe11	195.8571±11.83c	18.65714±1.18c	15.23±0.44e	94.57142±6.44b	0.4356±0.11b	0.187328±0.03b	0.533747±0.06b	1.294083±0.11c	73.36±4.85c	22.54±1.16d
Pe12	189.4632±10.38bc	18.63571±1.22c	18.12±0.82f	93.15466±6.73b	0.4894±0.13c	0.183081±0.04b	0.518322±0.05b	1.131667±0.10c	78.24±4.91d	22.68±1.28d

Values are presented as means ± SD. Means in a column with different letters are significantly different (*P* < 0.05). Mn, material number.

Statistical data analysis of cross-section anatomy (Table 2) shows that the lamina thickness (LT) increased from Pe1 to Pe8, and reached a maximum (0.5914 mm) in the material of Pe8, then decreased from Pe9 to Pe12 but maintained a higher thickness level. Ratio of epidermal cell thickness to lamina thickness (REL) shows an irregular distribution, and the lower REL was in the young leaves (Pe1) and the largest thickness in Pe8. Ratio of palisade tissue to lamina thickness (TCR) shows a similar change trend with LT, and arrived at a maximum ratio in the materials of Pe9 and Pe10.

### Epidermal micromorphological characteristics

**Fig 4:**
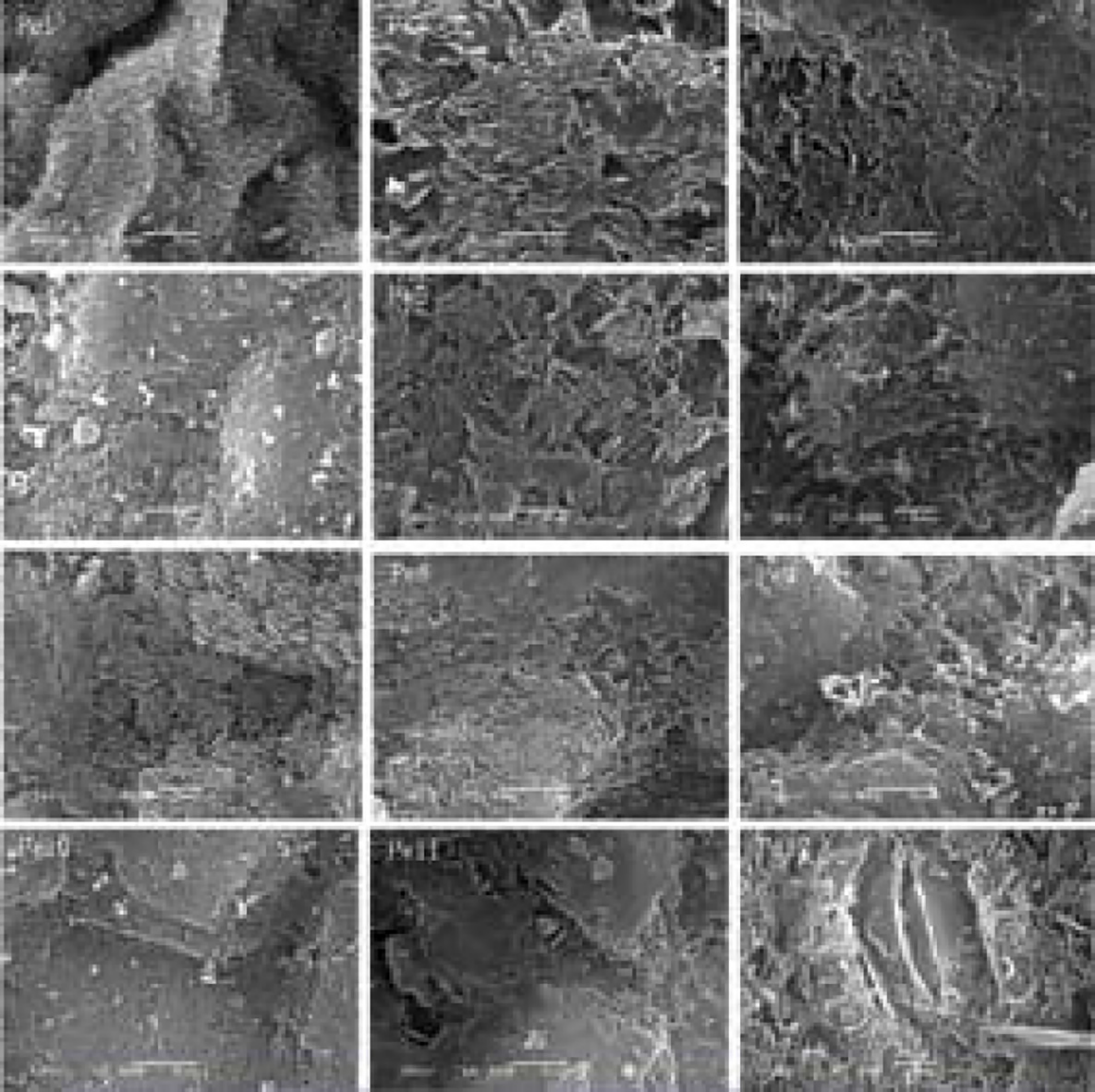
Morphological characters of epicuticular wax crystals on the adaxial epidermis of *Populus euphratica* heteromorphic leaves from Pe1 to Pe12. Scale bars: Pe1–Pe12 = 5 μm.

Thick lamellar epicuticular wax crystals were observed on the surface of the leaves (Fig 4). Wax crystals were often coalesced to form platelet-like structures or cloud-layer structures, and the cloud-layer wax increased from Pe1 to Pe12. Apart from Pe1 and Pe2, the cuticular wax coverage (CWC) on the leaf surface achieved more than 90% in the materials, and the cuticular wax thickness (CWT) increased from Pe1 to Pe12 (Table 2). The thickness was more than 1 μm from Pe6 to Pe12.

**Fig 5:**
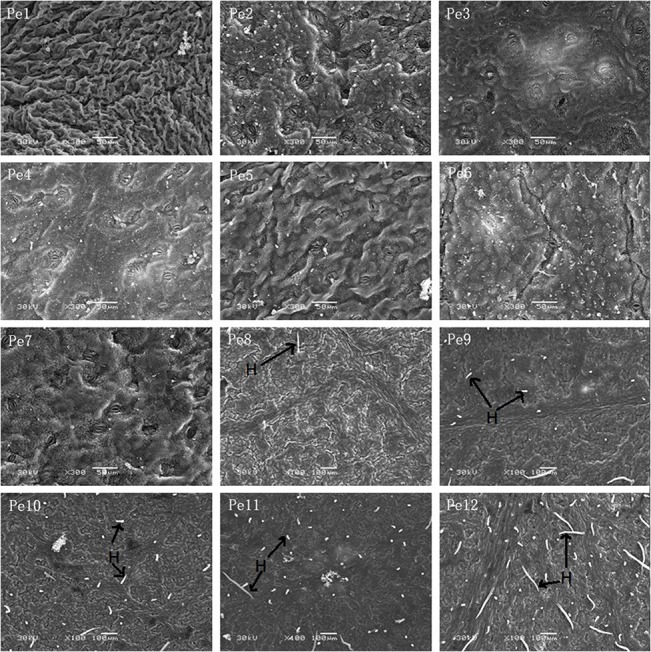
Trichomes on the adaxial epidermis of *Populus euphratica* heteromorphic leaves from Pe1 to Pe12. Scale bars: Pe1–Pe7 = 50 μm, Pe8–Pe12 = 100 μm. H, leaf hairs.

From Pe1 to Pe7, there were no trichomes on the epidermis of *P*. *euphratica* heteromorphic leaves (Fig 5). Trichomes appeared on both adaxial and abaxial surface from Pe8, and the trichome coverage (TC) increased quickly from Pe8 to Pe12, reaching a high coverage at 18.12% in Pe12 (Table 2).

**Fig 6:**
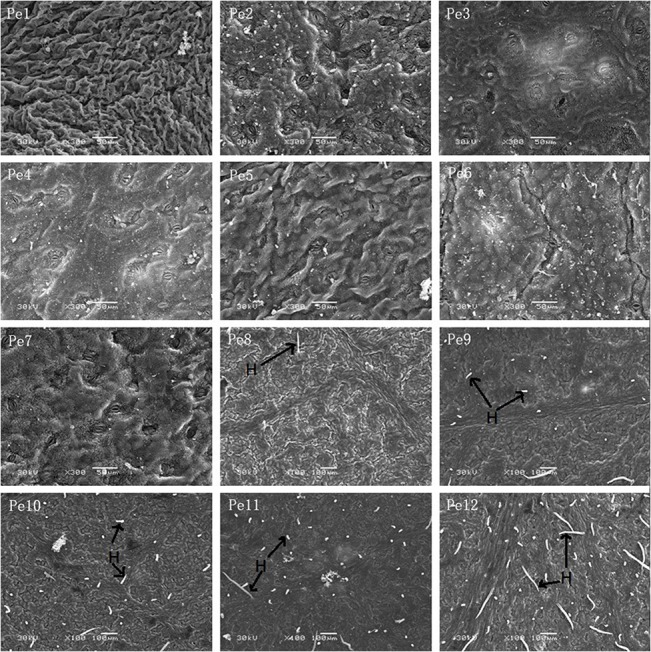
Morphological characteristics of stomata on the epidermis of *Populus euphratica* heteromorphic leaves from Pe1 to Pe12. Scale bars: Pe1–Pe12 = 5 μm.

Stomata were found on both abaxial and adaxial surfaces of all *P*. *euphratica* heteromorphic leaves. Dominate stomatal type is paracytic, of largely elliptical or oval-shaped (Fig 6). Stomata size (SS) increased from Pe1 to Pe12, but stomata density (SD) had no significant change from Pe2 to Pe10, except lower SD in the young leaves of Pe1. (Table 2). Epicuticular wax crystals were present in the epistomatal guard cells as well as on the surrounding epidermis.

### Chloroplast ultrastructural characteristics

**Fig 7:**
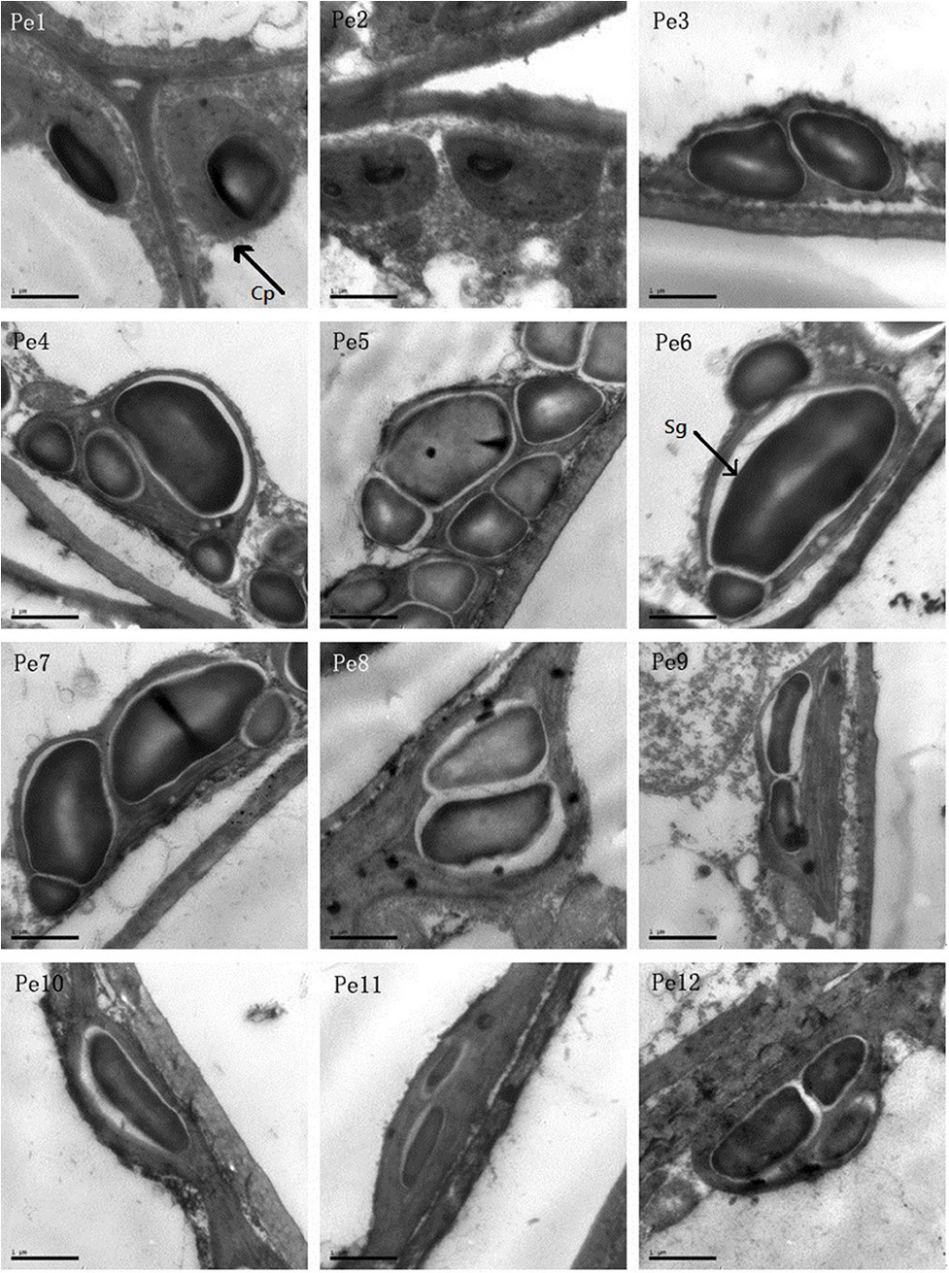
Chloroplast ultrastructural characters in the mesophyll cells of *P*. *euphratica* heteromorphic leaves from Pe1 to Pe12. The images are shown in ×30,000 magnification, scale bars = 1 nm. Cp, chloroplast; Sg, starch grain.

Chloroplast ultrastructure is illustrated in Fig 7. In Pe1 and Pe2, the chloroplasts are spherical or ellipsoidal. From Pe3 to Pe12, the chloroplasts are elongated and become fusiform shaped. The number and size of chloroplast and starch grains increased from Pe3 to Pe8. The chloroplasts are nearly full of starch grains from Pe3 to Pe8, and the leaf developing process increased the width of chloroplast due to the large size of starch grains. However, the number and size of starch grains decreased from Pe9 to Pe12.

### Hierarchical clustering analysis

**Fig 8:**
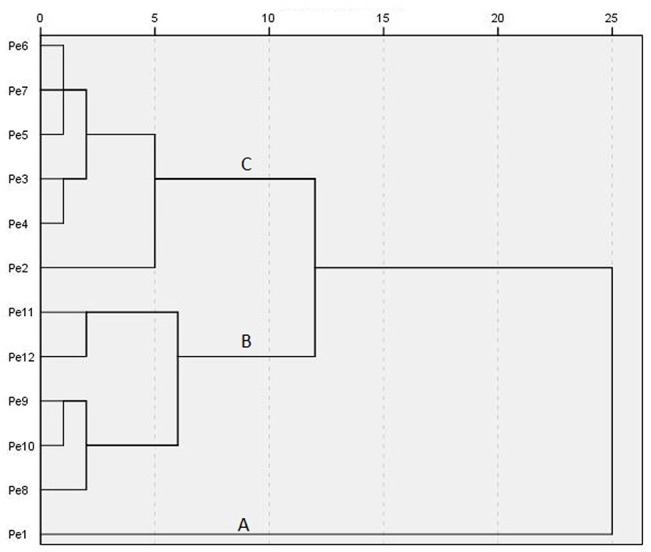
HCA dendrogram of *Populus euphratica* heteromorphic leaves.

The results obtained from the following hierarchical cluster analysis (HCA) are presented as a dendrogram (Fig 8), with three well-defined clusters. HCA involves a measurement of similarity between objects to be clustered, and samples with the maximum similarities were clustered preferentially [24]. The first cluster (A) consists of Pe1 alone because of young leaves with the lowest levels of all trait values (Table 2). A second cluster (B) is clearly discernible which is composed of Pe8 to Pe12. These samples are associated with high TC, CWT, NC and NM. A third cluster (C) includes Pe2 to Pe7, which represent the developing process of heteromorphic leaves. Thus, we believe that there are three functional groups of leaves in the development of heteromorphic leaves, young (Pe1), developing (Pe2 to Pe7) and mature leaves (Pe8 to Pe12).

### Leaf functional traits

Ranking of subordinate function values (Table 3) of heteromorphic leaves show that Pe1 has the lowest ability to stress resistance and Pe11 has the highest ability. This is consistent with the results of HCA in which Pe1 samples cluster alone as young leaves and Pe11 is in the mature leaves. The ability of stress tolerance was increased in the development of *P*. *euphratica* heteromorphic leaves from Pe1 to Pe12. Thus, the mature group with highest subordinate function values shows a higher stress resistance under arid desert conditions.

**Table 3:** Ranking of drought resistance in the development of *Populus euphratica* heteromorphic leaves based on subordinate function values.

Mn	SD	SS	TC	CWC	LT	REL	TCR	CWT	NC	NM	Mv	Rn
Pe11	1	0.925882	0.840508	0.862071	0.28663	0.616722	0.621311	1	0.852479	0.993827	0.799943	1
Pe12	0.822392	0.922671	1	0.691082	0.532967	0.546267	0.527045	0.862728	1	1	0.790515	2
Pe9	0.623017	0.901264	0.504415	0.891257	0.618132	0.591821	0.991108	0.990069	0.758464	0.821869	0.769142	3
Pe10	0.523825	1	0.747241	1	0.591575	0.098889	1	0.908157	0.783555	0.891534	0.754478	4
Pe8	0.571431	0.985824	0.19702	0.775864	1	0	0.54907	0.855859	0.694377	0.761023	0.639047	5
Pe6	0.341272	0.726791	0	0.827756	0.696886	0.5035	0.712576	0.881251	0.521765	0.626984	0.583878	6
Pe7	0.345239	0.72144	0	0.569286	0.639194	0.628235	0.707675	0.813143	0.535067	0.669312	0.562859	7
Pe5	0.444294	0.592993	0	0.983088	0.537546	0.262691	0.616123	0.526694	0.468863	0.558201	0.499049	8
Pe4	0.642858	0.483814	0	0.879314	0.598901	0.696549	0.10757	0.533526	0.40266	0.548942	0.489413	9
Pe3	0.519842	0.382127	0	0.620692	0.328755	1	0.567581	0.727567	0.307739	0.419312	0.487361	10
Pe2	0.540636	0.287934	0	0.034484	0.326923	0.413338	0.603472	0.482674	0.244861	0.118166	0.305249	11
Pe1	0	0	0	0	0	0.266556	0	0	0	0	0.026656	12

Mn, material number; Mv, mean value; Rn, Ranking number.

## Discussion

In this study, quantitative values of leaf epidermal micromorphology and mesophyll structures of twelve heteromorphic leaves were selected from one *P*. *euphratica* tree. Because some of the selected mature trees where uncertain as to their age and the values of mesophyll structures varied in the same types of heteromorphic leaves of different trees. But these values have the same changed trend in different *P*. *euphratica* trees. In order to show significant changes among twelve heteromorphic leaves, all values of leaf epidermal micromorphology and mesophyll structures were selected from many leaves of the same types in one *P*. *euphratica* tree and not of the mean values from all selected trees.

Leaf cross-section anatomical characteristics show that *P*. *euphratica* is a typical xerophyte. The leaves are isobilateral, with only closely-arranged palisade tissues in the mesophyll structure between the upper and lower epidermal cells. Under desert conditions, it is believed that the high palisade packing of the thick glabrous leaves protects them from rapid wilting during water stress [25]. Also, results show that lamina thickness (LT), ratio of epidermal cell thickness to lamina thickness (REL) and palisade tissue to lamina thickness (TCR) increased from Pe1 to Pe8. A significant positive correlation exists between thicknesses of the leaf and epidermis. Epidermal cells may be considered as a buffer layer and may contribute to the reflectance and absorption properties. The relatively thick leaves increase the absorption of the near infrared radiation by their thick epidermal cells before the radiation transmits to the internal mesophyll of the leaf in glabrous leaves, and thus protect leaves from injury [25]. These xeromorphic characteristics have been reported in previous studies [13, 18], and not emphasize in the present research.

A new discovery is numerous crystal idioblasts and mucilage cells that fill the palisade tissues in dentate mature leaves. Crystal formation is considered to be associated with a variety of proposed functions, including calcium regulation, ion balance, plant defense and the provision of tissue structural support [26, 27]. More than 215 plant families possess CaOx crystals which have been observed in virtually all organs, tissues and cell types [26]. Some studies have shown that CaOx crystals are more numerous in arid zone species [28]. Crystal idioblasts have a higher osmotic potential and strong water absorption capacity in desert or xeric plants [29]. They can absorb and store water under better environmental water conditions, and provide a relatively humid environment to their surrounding cells under water stress. This process helps to improve the drought resistance of desert plant. Mucilage cells are observed only in mature leaves of adult plants and are characterized as enucleated, lacking cytoplasm, vacuoles, and other cytoplasmic materials [30]. Zimmermann et al. [31, 32] found that mucilage apparently plays a crucial role in the water supply of apical leaves, not only of high-salinity-tolerant but also in some low-salinity-tolerant trees. Mucilage-mediated moisture uptake by apical leaves from the atmosphere may be as important as (or more important than) water uptake by the roots. This is the process termed “reverse transpiration” by plant physiologists [33, 34]. Also, mucilage formation is apparently enforced by water stress [35]. Thus, from our results, we can conclude that mature *P*. *euphratica* leaves can effectively defend against drought stress through the protection of leaf xerophytic anatomy, regulate the internal water situation of the microenvironment by crystal idioblasts, and facilitate moisture uptake from the atmosphere through mucilage cells in the mesophyll.

The epidermis of desert plant leaves is often ornamented with a thick waxy cuticle. In this study, we found that the epicuticular wax coverage and thickness increased from Pe1 to Pe12 in the development of *P*. *euphratica* heteromorphic leaves. Cuticular waxes and cutin form the cuticle, a hydrophobic layer covering the aerial surfaces of land plants, acting as a protective barrier against environmental stress [36, 37]. In many plant species, an amorphous film of epicuticular material forms a smooth wax surface, while wax crystals protruding from this film create a microscopically rough surface on others [38]. The epicuticular wax forms the outermost surface of the plant, and is thus of special importance in interactions with the biotic and abiotic environment [39]. This cuticular covering is considered as a mechanism to minimize transpirational water loss through the epidermis and trichomes [25].

One of the striking features of many desert plant leaves is the presence of pubescence (hairs), and this has long been positively associated with arid climates [40]. Leaf pubescence is thought to possibly increase the thickness of the leaf boundary layer [41, 42] and reduce leaf absorptance resulting in a reduced heat load, and as a consequence lower leaf temperatures and transpiration rates [43]. Leaf hairs are adaptive features of plants to arid conditions [44]. In our study, leaf hairs were observed on the epidermis of dentate broad types of leaves and increased from Pe8 to Pe12, although with only a few hairs on Pe8 epidermis. This systematic study on the development of micro-morphological epidermis has found inconsistent result compared to the observation of the three typical diversiform-leaves by Li and Zheng [18]. They believed that there was no cilium on the *P*. *euphratica* leaf epidermis only covered with wax. Obviously, the three heteromorphic leaves they selected can’t represent all leaf types of *P*. *euphratica*. It is necessary and important to analyze all leaf types and their quantitative characteristics in the present study. An advantage of leaf pubescence is that it allows a leaf temperature much lower than air temperature. As a result, leaf temperatures are near the temperature optimum for photosynthesis and high, potentially lethal leaf temperatures are avoided [40]. In other words, under arid conditions and/or high air temperatures, leaves of *P*. *euphratica* have a higher rate of photosynthesis by being pubescent than by not being pubescent. Combining the results of epicuticular wax coverage and thickness, it is suggested that the dentate broad leaves (from Pe8 to Pe12) with thick cuticular wax and trichomes of *P*. *euphratica* trees play important roles in protection from high temperatures, reducing transpiration and enhancing adaptability under arid desert conditions. A thick waxy cuticle and trichomes restrain non stomatal water loss from the epidermis of dentate mature leaves in *P*. *euphratica* tree.

Stomata are pores or openings for gas exchange in the epidermis of plants. The main control of water movement in plants is provided by stomata [45]. Features of stomatal architecture consist of stomatal density, pore dimension, shape, and associated subsidiary cells. Their variations are suggested to profoundly affect gas exchange and the pathways of water inside the leaf [46]. Paracytic stomata dominate in *P*. *euphratica* heteromorphic leaves, usually elliptical or oval-shaped. Stomata size (SS) increased from Pe1 to Pe12, but stomata density (SD) had no significant change from Pe2 to Pe10. According to Brown and Escombe [47] and Sayer [48], the rate of diffusion of water vapour through smaller pores is more than through larger ones and the rate of diffusion is more nearly proportional to the perimeter rather than its area. Thus, the rate of water vapour diffusion through the stomatal pores decreased from Pe2 to Pe10 owing to increased pore perimeter and almost constant stomatal density. The result also indicates that there is a potential higher rate of gas exchange in the mature leaves with higher SD and SS under arid environments. Photosynthetic rate was higher in ovate leaves than in lanceolate leaves [9, 10, 17], and the dentate broad ovate leaves had more effective energy allocation strategy [21]. Zheng et al. [17] proved that the average values of the diurnal photosynthetic rate of these leaves are different, which decrease from broad ovate, dentate broad ovate leaves to lanceolate. Analysis of chlorophyll fluorescence parameters also showed that photochemical efficiency of PSII is higher in dentate broad-ovate leaves of old trees [20]. This is consistent with chloroplast observation in *P*. *euphratica* heteromorphic leaves, where the number and size of chloroplast and starch grain increased from Pe3 to Pe8. Chloroplasts are cytoplasmic organelles and the sites of photosynthesis in eukaryotic cells [49]. Chloroplast structure determines photosynthetic capacity of leaf cells. Vanden Driessche [50] found that *Acetabularia mediterranea* has the greatest photosynthetic capacity with elongated chloroplast shape during the middle of the light period, but the chloroplast are spherical during the middle of the dark period. On the other hand, an increased chloroplast number could achieve a higher photosynthetic rate at elevated CO_2_ concentrations [51]. From these results, we can conclude that there was a higher photosynthetic rate in Pe5 to Pe8 of *P*. *euphratica* heteromorphic leaves under well watered conditions. Also, a large number of elongated chloroplasts indicate that there is a potential higher photosynthetic capacity in materials from Pe9 to Pe12 under arid conditions.

HCA based on maximum similarities shows that there are three groups of *P*. *euphratica* heteromorphic leaves: young (Pe1), developing (Pe2−Pe7) and mature leaves (Pe8−Pe12). From the results of chloroplast ultrastructural characters and subordinate function values of *P*. *euphratica* heteromorphic leaves, the developing leaves have maximal photosynthetic activity, and the mature leaves have maximal protective ability from adverse environments and achieve high photosynthesis efficiency under arid conditions. Thus, the main function is differentiated in the heteromorphic leaves of one *P*. *euphratica* tree in a natural habitat, and we can deduce that it is different in the proportion of different types of heteromorphic leaves in different ages of *P*. *euphratica* trees. The higher age of the tree, the higher proportion of mature leaves that evolved.

## Conclusions

In order to improve the overall resistance of *P*. *euphratica* trees to adverse environmental conditions, different types of heteromorphic leaves have evolved. There are three types of functional groups, young, developing and mature leaves. Photosynthetic activity and stress resistance increased during the developing process from Pe2 to Pe8 of heteromorphic leaves. The maximum photosynthetic activity was reached in leaves of Pe5 to Pe8. However, the maximum adaptability to drought tolerance was exhibited in Pe9 to Pe12, which are the developed mature leaves. Thus, Pe8, with a dentate rhombic leaf form and the first appearance of trichomes, is the transition point between developing and mature leaves. The mature leaves can effectively defend against drought stress by high photosynthesis and stress tolerance under arid desert conditions, through the protection of leaf xerophytic anatomy, regulation of the internal-microenvironment water situation associated with mucilage and crystal idioblasts in the mesophyll, and assistant defense by leaf epidermis appendages such as cuticular wax and trichomes. Our results confirm that leaf function is differentiated during the evolutionary process of heteromorphic leaves. This process is the result of the plant’s adaptation to the environment. Thus, we can deduce that there is completely different proportion of leaf types in *P*. *euphratica* trees correlated with different ages. The older the age of *P*. *euphratica* trees, the higher proportion of mature leaves that evolved in response to adverse environmental conditions.

## Supporting Information

S1 Fig Sketch of Heihe River and the location of the sampling site (*) in Ejina (Adopted from Cao *et al*. [22]). (JPG)

S2 Fig *Populus euphratica* forest and its natural habitat in the National Natural Reserve of *P*. *euphratica* in the Ejina Oasis at the lower reaches of the Heihe River. (JPG)
