# Correction: Epidermal Micromorphology and Mesophyll Structure of *Populus euphratica* Heteromorphic Leaves at Different Development Stages

**DOI:** 10.1371/journal.pone.0141578

**Published:** 2015-10-23

**Authors:** Yubing Liu, Xinrong Li, Guoxiong Chen, Mengmeng Li, Meiling Liu, Dan Liuy

The image for Fig 4 is corrected for improved readability. Please see the correct Fig 4 here.

**Fig 4:**
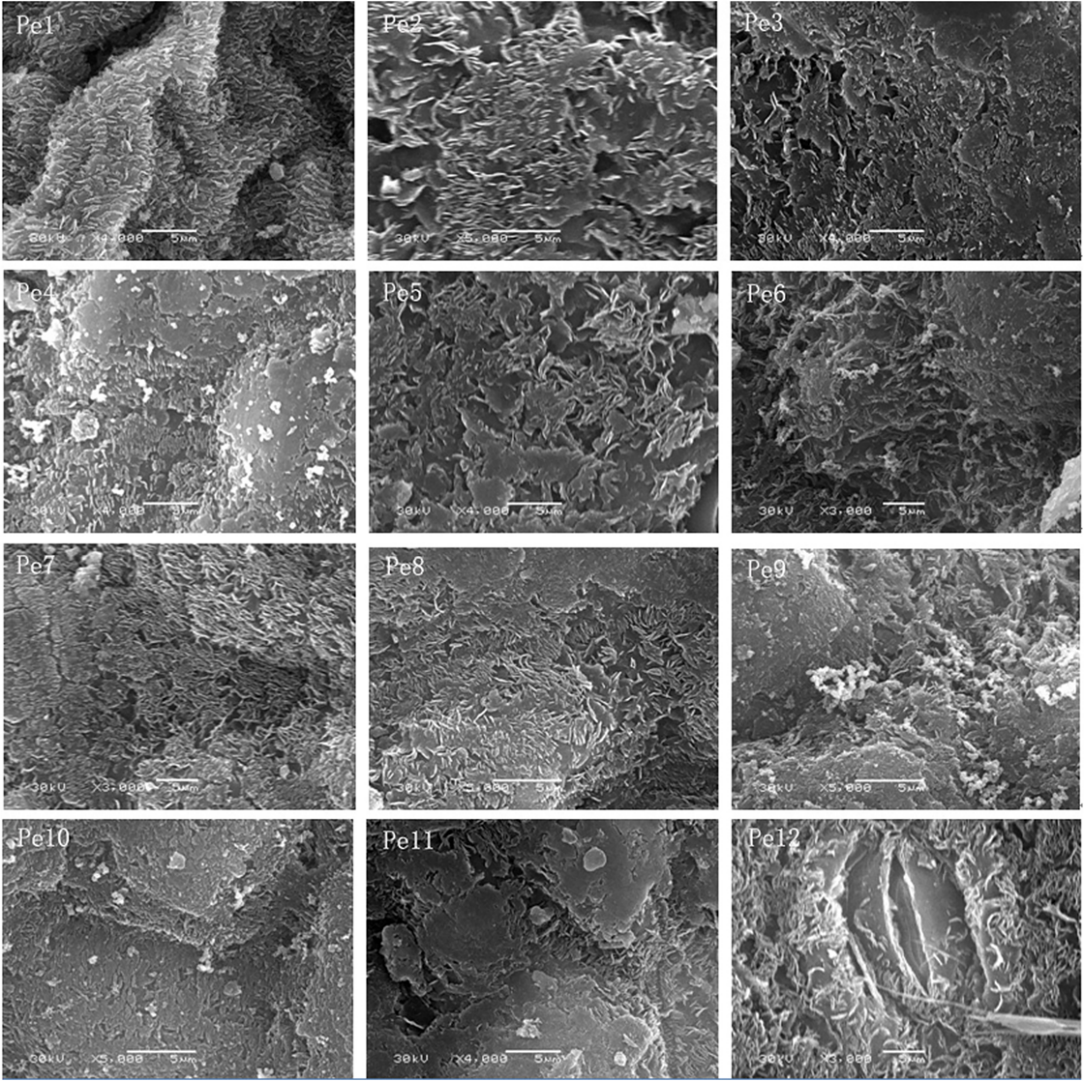
Morphological characters of epicuticular wax crystals on the adaxial epidermis of *Populus euphratica* heteromorphic leaves from Pe1 to Pe12. Scale bars: Pe1–Pe12 = 5 μm.

The image for Fig 6 is incorrect. Fig 6 is a duplicate image of Fig 5. Please see the correct Fig 6 here.

**Fig 6:**
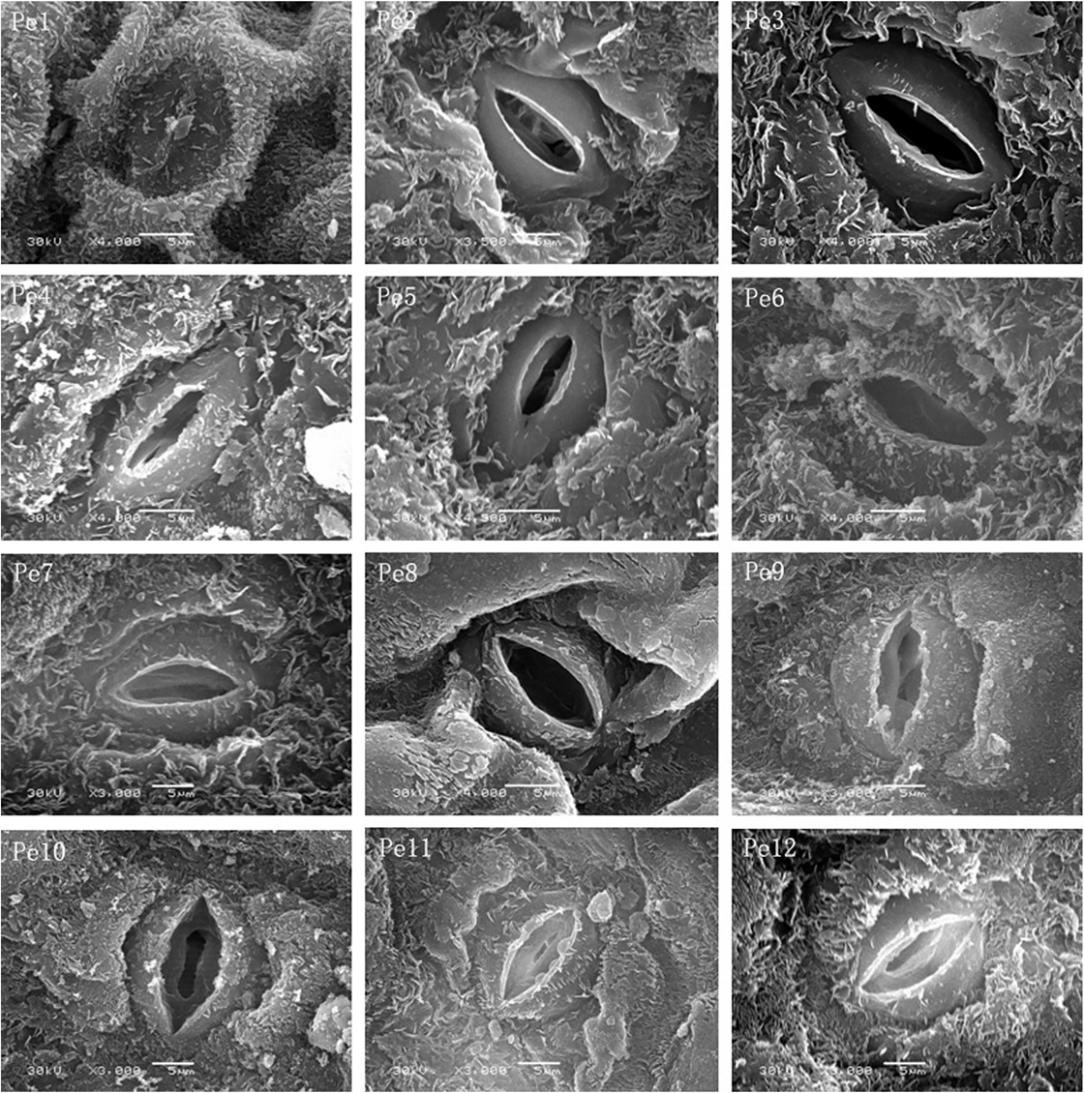
Morphological characteristics of stomata on the epidermis of *Populus euphratica* heteromorphic leaves from Pe1 to Pe12. Scale bars: Pe1–Pe12 = 5 μm.
