# Supplementary figures and images for: Epidermal Micromorphology and Mesophyll Structure of Populus euphratica Heteromorphic Leaves at Different Development Stages
Source: PLoS One. 2015 Sep 10;10(9):e0137701. doi: 10.1371/journal.pone.0137701 (PMC4565707; doi:10.1371/journal.pone.0137701)

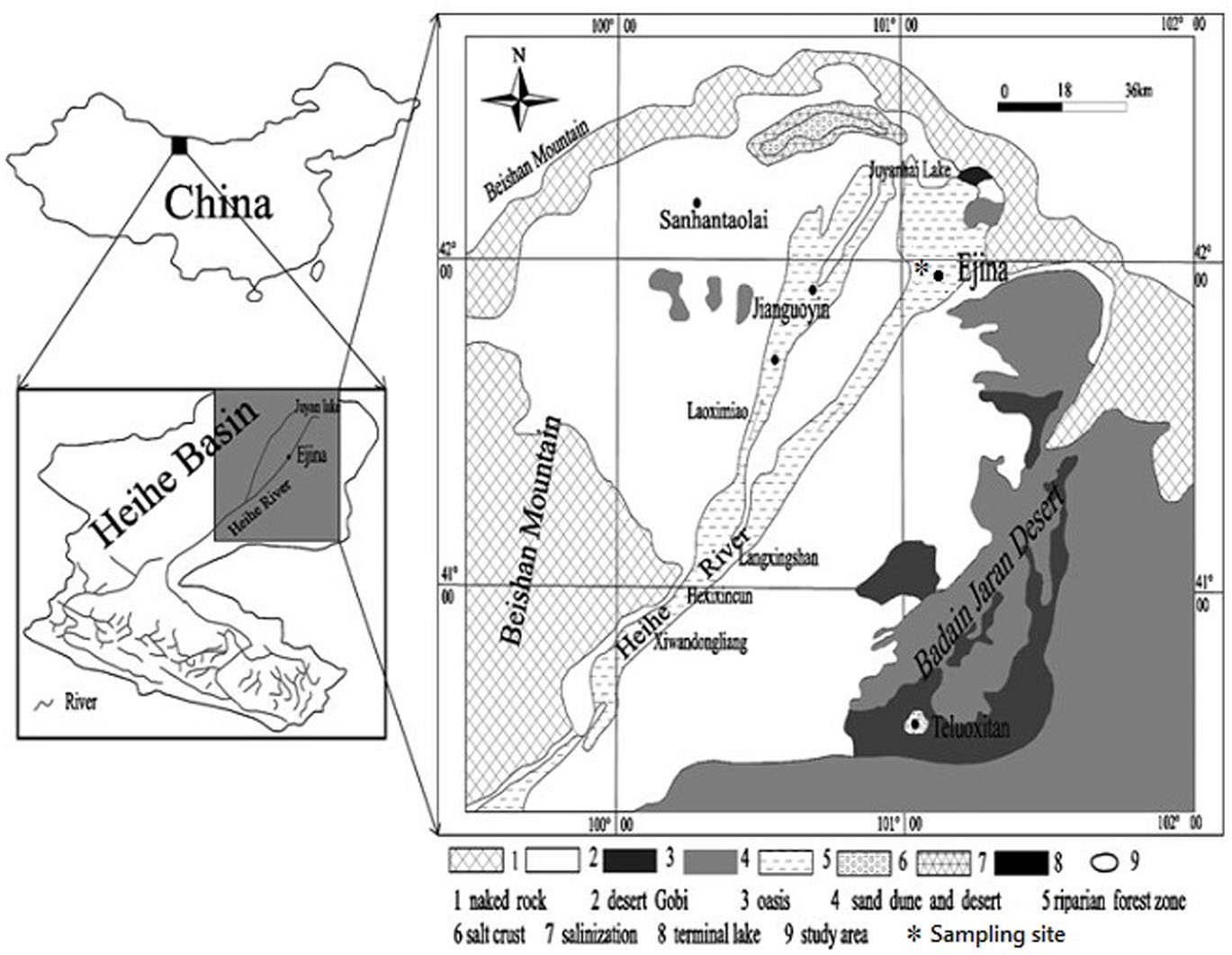

Supplement: S1 Fig — (JPG) [file pone.0137701.s001.jpg]

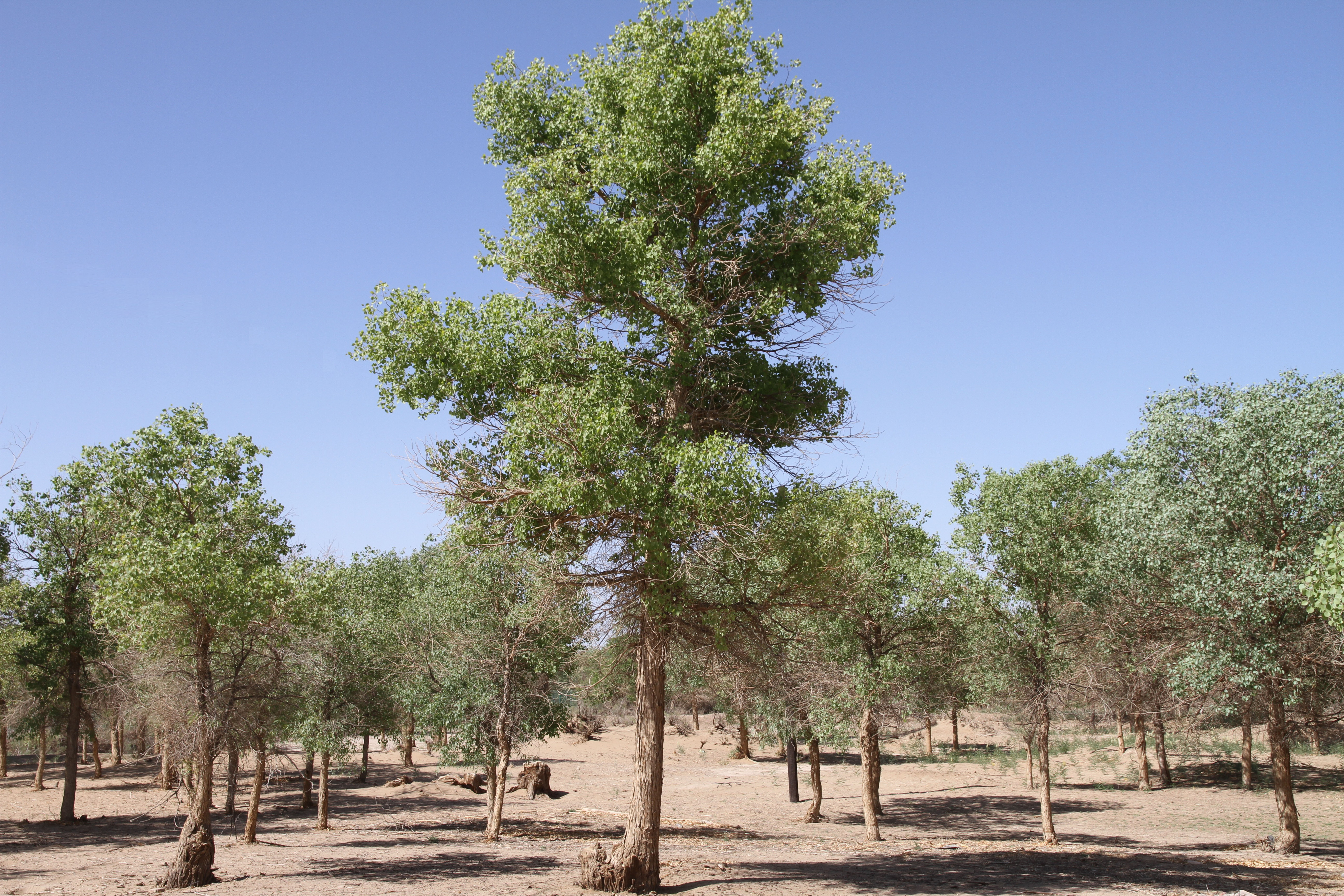

Supplement: S2 Fig — (JPG) [file pone.0137701.s002.jpg]
